# Mechanical Properties and Cushioning Effectiveness of FPUF-EPS Combination Materials

**DOI:** 10.3390/ma16216886

**Published:** 2023-10-27

**Authors:** Zexiong Zhang, Weizhou Zhong, Jiaxing Li, Jingrun Luo

**Affiliations:** 1 Department of Modern Mechanics, University of Science and Technology of China, Hefei 230026, China; 2 Shock and Vibration of Engineering Materials and Structures Key Laboratory of Sichuan Province, Mianyang 621999, China

**Keywords:** combination materials, cushioning effectiveness, numerical analysis, flexible polyurethane foam, expanded polystyrene foam

## Abstract

Based on flexible polyurethane foam (FPUF), which is reversible after compression, and expanded polystyrene foam (EPS), which has a high cushioning energy absorption capacity, the parallel and series combinations of FPUF and EPS are provided. According to experimental data of FPUF and EPS uniaxial compression large deformation, the mechanical properties and cushioning effectiveness of the FPUF-EPS combination materials with different structural scale parameters were investigated by theory analysis and finite element simulation. The mechanical response and the cushioning effectiveness influencing factors of FPUF-EPS parallel (FE-P) and FPUF-EPS series (FE-S) combination materials under single compressive load, single-impact load, and multiple compressive loads were obtained. The differences in mechanical properties and cushioning effectiveness of FE-P, FE-S, FPUF, and EPS are analyzed. The influence law of structural scale parameters and load strength on the mechanical properties and cushioning effectiveness of FE-P and FE-S is provided. It indicates that the cushion properties of combination materials should be adjusted to satisfy product protection requirements. It is beneficial for the design optimization of cushioning and packaging protection.

## 1. Introduction

Cushioning energy-absorbing materials have a wide application in the field of packaging protection. The stress–strain curve of suitable cushioning materials should have the characteristics of a wide plateau stress area, little fluctuation in stress amplitude under large deformation conditions, and a large compaction strain. The porous material is typical cushioning material with wide plateau stress, which consists of random or periodically distributed pores in the microstructure, and the cells are surrounded by a three-dimensional grid composed of pores and cell walls. The microstructure of porous material induces plateau stress under large deformation conditions [1].

There are many natural porous materials, such as wood, bones, sponges, etc. Some of these materials have pores of random size and shape in their microstructure. Some porous materials have periodic pore structures, such as honeycombs with hexagonal cells. Based on the variety of natural porous materials, synthetic porous materials have been developed for certain requirements. Polymeric foams were synthesized by adding blowing agents to polymers [2,3]. Blowing agents were also used in matrix materials to synthesize various kinds of porous materials. Due to the high-strength mechanical properties of metallic materials, various types of metallic foams have been produced. Aluminum foams [4,5,6,7] and titanium foams [8,9] were used widely for their excellent properties. Non-metallic low-density porous materials are usually used as cushioning, energy-absorbing parts in industrial product packaging, such as plastic foam, silicone rubber foam, etc. It indicates that the effective energy dissipation and absorption of cushioning materials play an important role in the integrity and safety of products.

Cushioning materials can be classified into single-use energy dissipation and multi-use energy storage according to the deformation reversibility. Single-use cushioning material dissipates kinetic energy to achieve the cushioning effect on the protected object by plastic deformation, and the energy transformation process is irreversible. For example, vehicle bumpers generally dissipate energy using plastic folds generated by thin-walled structures of lightweight metal [10]. Metal corrugated guardrails of highways and porous foam materials used in the packaging of electrical products are taken as single-use cushion materials [11,12,13]. Multi-use cushioning materials absorb external kinetic energy by elastic deformation energy storage. It can extend the loading time and weaken the peak load, which reduces the impact response of the protected object. The elastic deformation recovery is with the external loading decreasing. The accumulated elastic energy is released, such as the spring cushion used in pole vaulting and the rubber cushion of bumper cars.

Flexible polyurethane foam (FPUF) and expanded polystyrene foam (EPS) are commonly used as cushioning energy-absorbing materials, and they are usually used in impact cushioning and shockproof packaging. Foam materials have wide plateau stress, and the mechanical response can be characterized by three deformation stages. In the first stage of linear elastic deformation, the cell wall is with small bend deformation. In the second stage of the wide stress plateau, the stress increases slowly with strain. Much energy absorption is accomplished in this stage. In the third stage, the material enters densification deformation, which induces the cell units to be compressed completely, and the stiffness and stress levels increase dramatically [14,15,16]. The stress plateau of EPS is much higher than that of FPUF. It indicates that the energy absorption of EPS is much stronger than that of FPUF. Unlikely compressed non-reversible EPS, FPUF has a compressed reboundable performance. It means that EPS is an energy-dissipative cushioning material and can only be used once, while FPUF can be used repeatedly. FPUF has better cushioning protection in multiple loading conditions.

FPUF and EPS are widely used in the fields of manufacturing and industrial packaging. Many scholars have investigated the static and dynamic mechanical properties. Yang [17] and Scarfato [18] investigated the mechanical properties of FPUF using compression experiments and explored in detail the microstructure and composition effect on the properties. Gholami [19] systematically investigated the effects of cell size and relative density on the mechanical properties of FPUF and proposed a new micro-macro model to describe FPUF mechanical behavior. Song [15] tested EPS dynamic behavior using a modified split Hopkinson pressure bar to investigate the effect of strain rate on elasticity and early cell collapse response. Chen [16] investigated quasi-static and dynamic mechanical properties of EPS by Baldwin and material test machine. The compressive strength, tensile strength, elastic modulus, and energy absorption properties of two different density foams at different strain rates were provided by the tests. It indicates the mechanical properties are strain rate dependence. Guo [20] investigated the influence mechanism of microstructure on the mechanical and thermal properties of EPS specimens with different microstructures. Chuda-Kowalska [21] proposed a mixed experimental–numerical analysis method to test the mechanical properties of orthotropic foams. This method can reduce the number of tests and obtain the mechanical performance parameters that characterize orthotropic foam materials.

It Is difficult to achieve a superior energy-absorbing effect with traditional natural cushioning material for product packages in complex stress conditions. Combination structures of different cushion materials are adopted to obtain better cushioning protection performance in complex mechanical environments. For example, the combination of rigid and flexible materials is applied to reduce impact response induced by high-speed impact loading [22]. The combination of compressive reboundable and non-reboundable cushioning materials can not only maintain a high level of cushioning energy absorption capacity but also prolong the action time and weaken the load peak under multiple loading conditions. The combination cushioning structure can improve mechanical properties, such as impact resistance and cushioning energy absorption. It is applied widely in product protection, engineering reinforcement, and ballistic fields. Much literature has focused on the dynamic mechanical properties, energy absorption mechanisms, and strain rate effects of the combination structures of different materials in recent years [23,24,25,26,27]. Jung [23] investigated the strain rate sensitivity of Ni/Al hybrid foam metallic materials by quasi-static and split Hopkinson pressure bar dynamic experiments. Ballistic impact experiments of surface loading and point loading were accomplished. The difference in ballistic properties between pure and combination foams was evaluated. Zhao [24] investigated reverse penetration tests of aluminum foam sandwich panels under impact loading. It showed the high compressive stress distribution is located in a small region, and strain hardening of the aluminum foam occurred during the penetration process. Cantwell [25] tested the dynamic response of foam polymer and foam metal sandwich materials under low-velocity impact loading by drop hammer. The failure modes include face peeling, shear cracking, and inner fragmentation. Zenkert and Burman [26] found the stiffness and flexural strength of the sandwich panels increase significantly with the height of the V-shaped core, which brings small mass increases. Radford [27] investigated the impact response of sandwich beams of different combination modes. It indicated the sandwich beam composed of stainless steel tapered core and foam aluminum has a smaller impact resistance, and the combination composed of stainless steel corrugated core and foam aluminum alloy has a higher impact strength.

In summary, EPS has a high cushioning energy absorption capacity but is not reversible after compression and cannot be used repeatedly. FPUF has the ability to return after compression, but the cushioning energy absorption capacity is relatively poor. In order to improve cushion structure properties, the combination of EPS and FPUF (EPS-FPU) is provided to achieve a high level of cushioning energy absorption. It can not only prolong the action time but also weaken the impact force peak. The cushioning performance of the EPS-FPUF combination and the influencing factors are little paid close attention. Two EPS-FPUF modes, parallel combination (FE-P) and series combination (FE-S), are investigated to gain the mechanical properties and cushioning effectiveness influencing factors under single static compression, single-impact compression, and multiple compression conditions. Based on numerical simulations of the compression response of FE-P, FE-S, FPUF, and EPS, the cushioning effectiveness of four cushion structures is compared, and the influence of structural scale parameters on the cushioning effectiveness of FE-P and FE-S is also evaluated.

## 2. Materials and Methods

### 2.1. Model Analysis

**Figure 1:**
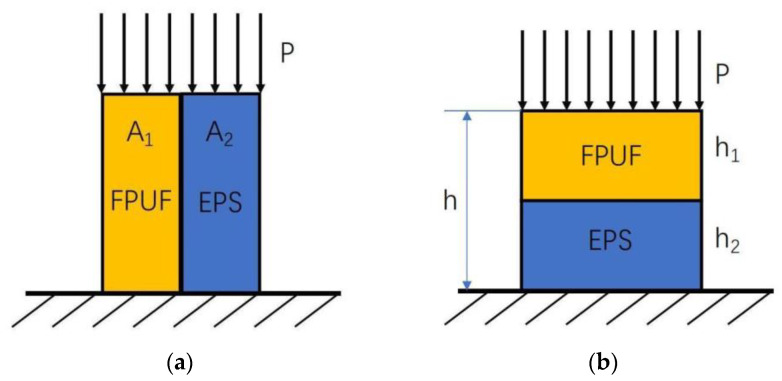
FPUF-EPS combination materials in parallel (**a**) and series (**b**).

Two combination modes of parallel combination and series combination are shown in Figure 1, respectively. For the FPUF-EPS parallel combination (FE-P) in Figure 1a, the loading area of the whole model is taken as *A*, the load area of the FPUF part is $A_{1}$, and the load area of EPS is $A_{2}$. The assembled force is taken as *P*, and the forces on FPUF and EPS are $P_{1}$ and $P_{2}$, respectively. The stress of FE-P is expressed as Equation (1). During the compression process, the deformations of the FPUF and EPS are coincident, and the stress of FE-P can be written as Equation (2). In the elastic deformation stage, the equivalent elastic modulus is written as Equation (3), and *E*_1_ and *E*_2_ are the elastic modulus of FPUF and EPS, respectively. According to the structure diagram of the FPUF-EPS series combination (FE-S) shown in Figure 1b, the thickness of FE-S is taken as *h*, and the thicknesses of FPUF and EPS are $h_{1}$ and $h_{2}$, respectively. When the external force is applied, the whole deformation is taken as *x*, which includes the deformations of $x_{1}$ and $x_{2}$ produced by FPUF and EPS, respectively. Based on theoretical analysis, the strain of the series combination material consists of two parts. As FPUF and EPS have the same pressure, the strain can be expressed as Equation (4). The equivalent strain of FE-S can be simplified using Equation (5). According to the different proportions of FPUF and EPS in the FE-S material, the equivalent strain of FE-S can be calculated for elastic compression deformation.

(1) $$\sigma =\frac{P}{A}$$

(2) $$\sigma =\alpha \sigma_{1}+\beta \sigma_{2}\;\left(\;\alpha =\frac{A_{1}}{A},\;\beta =\frac{A_{2}}{A},\;\sigma_{1}=\frac{P_{1}}{A_{1}},\;\sigma_{2}=\frac{P_{2}}{A_{2}}\;\right)$$

(3) $$E=\alpha E_{1}+\beta E_{2}$$

(4) $$\varepsilon =\frac{x}{h}=\frac{x_{1}}{h}+\frac{x_{2}}{h}=\frac{h_{1}}{h}\frac{x_{1}}{h_{1}}+\frac{h_{2}}{h}\frac{x_{2}}{h_{2}}$$

(5) $$\varepsilon =\mu \varepsilon_{1}+\tau \varepsilon_{2}\left(\mu =\frac{h_{1}}{h},\;\tau =\frac{h_{2}}{h}\;\right)$$

The combinations of FPUF-EPS materials in parallel and series exhibit different responses at the same compressive loading condition. For the different structural scale parameters of FPUF and EPS, the combination of FPUF-EPS material (FE) produces different mechanical properties and cushioning effectiveness. In order to analyze the structural scale parameter effect on mechanical properties of the FPUF-EPS combination material, several combination materials of FE0 (EPS), FE0.2, FE0.4, FE0.5, FE0.6, FE0.8, and FE1 (FPUF) were designed for analysis, as shown in Table 1.

### 2.2. Numerical Simulation

#### 2.2.1. Finite Element Model

**Figure 2:**
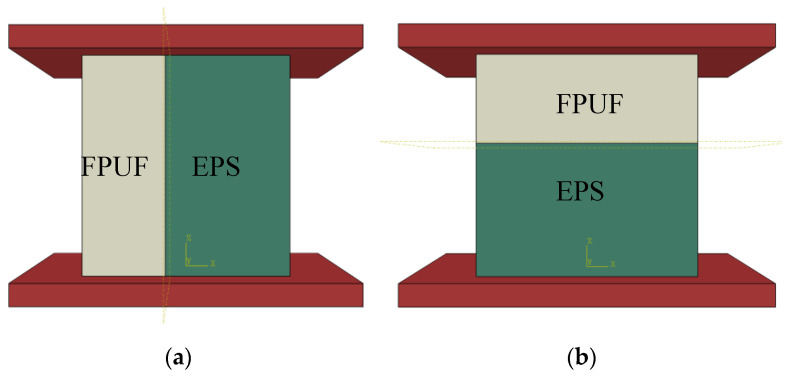
Three-dimensional models of FPUF-EPS combination materials in parallel (**a**) and series (**b**).

A full-size FPUF-EPS combination material model of 50 × 50 × 50 mm was established in Figure 2. The upper impact table and the lower base plate are made of steel. The density is 7800 kg/m^3^, and the elastic modulus is 200 Gpa. The impact table size was 80 × 80 × 20 mm. The density of FPUF material is 77.8 kg/m^3^, and the density of EPS material is 43.5 kg/m^3^. In the numerical simulation, the contact mode is defined as “general contact”, and the contact properties are set to “hard contact” in the normal direction and “frictionless contact” in the tangential direction.

#### 2.2.2. Material Model

The crushable foam model can better simulate the compressible foam. The model default material deformation cannot be immediately recovered, so it is equivalent to the plastic deformation of the material and used in conjunction with the linear elasticity material model. The crushable foam model in the rebound stage of the displacement is very small and generally can only be used for compression of non-responsive materials. The hyperfoam material model can be applied to elastomeric foams with low density, compression recovery, and strain rate sensitivity. Therefore, the FPUF finite element simulation uses the hyperfoam model, which requires the input of the material’s uniaxial tensile or compressive nominal stress–strain curves.

**Figure 3:**
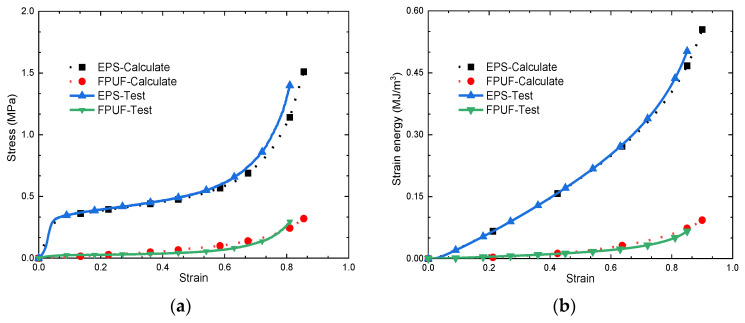
Comparison of stress–strain curves (**a**) and strain energy curves (**b**) of FPUF and EPS obtained from experimental tests and numerical calculations.

In order to establish the material model of the FPUF-EPS combination materials, we performed uniaxial compression large deformation experimental tests on a neat FPUF and a neat EPS, which are plotted in Figure 3. For the FPUF part, the FPUF material is reboundable after compression, so the hyperfoam material model (characterizing compression recoverable strain rate-sensitive elastomeric foam) is used to define the material properties, and the nominal stress–strain curves for uniaxial testing of FPUF are imputed directly. The stress–strain curves of different strain rates are imputed when considering strain rate dependence. The crushable foam model is suitable for compressible foam, such as EPS material. The resilient displacement is little and can be neglected after compression loading. The elasticity modulus of EPS for the linear phase is taken as 6.16 MPa, and Poisson’s ratio is 0.01. The data for the plastic phase were obtained based on the stress–strain curves from the uniaxial compression test of EPS. Firstly, the nominal stress–strain curve obtained from the experimental test was converted into the true stress–strain curve, then the plastic stress and strain were calculated. Finally, the yield stress and plastic strain data are inputted into the finite element software to complete the definition of the elastic–plastic material model.

#### 2.2.3. Boundary Conditions

In order to comprehensively study the cushioning performance characteristics of FPUF-EPS combination materials in packaging protection. Based on the actual working conditions, we chose to analyze the cushioning effectiveness of FPUF-EPS combination materials under three conditions, namely, single compression, single impact, and multiple compression. Under single compression, the impact plate mass is 1 kg, and the uniform moving speed is 1 m/s. Under single impact, the impact plate mass is 1 kg, and the initial velocities are 3 m/s (4.5 J), 5 m/s (12.5 J), and 10 m/s (50 J) in the simulations. Under multiple compressions, the loading condition is the boundary displacement controlling. The multiple compressive loadings are applied by four loading steps. The deformation is compressed to 0.1 and then released in the first loading step. The deformation strain of 0.2, 0.4, and 0.8 is applied and released in the subsequent loading steps, respectively.

#### 2.2.4. Model Validation

In order to verify the accuracy of the material model parameters in the finite element analysis, a comparison between numerical simulation and experimental results under uniaxial compression with large deformation is performed. The stress–strain curves and strain energy curves (energy absorption per unit volume) of FPUF and EPS obtained by numerical simulation are consistent with the results of tests in Figure 3. It indicates that the numerical simulation can accurately simulate the deformation of FPUF and EPS during large compression deformation.

## 3. Cushioning Effectiveness of FPUF-EPS under Single Quasi-Static Compression Loading

### 3.1. Cushioning Effectiveness of FE-P

**Figure 4:**
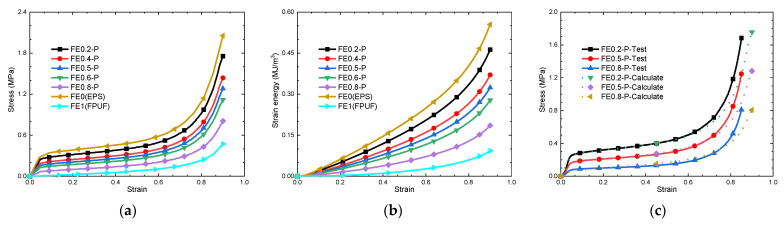
Stress–strain curves (**a**), strain energy–strain curves (**b**), and comparison of stress–strain curves of FE-P obtained from experimental tests and numerical simulations (**c**).

The stress–strain curves and strain energy (energy absorption per unit volume)–strain curves of FE-P obtained by numerical simulation are shown in Figure 4. It can be seen that the characteristics of the stress–strain curves of FE-P under single uniaxial compressive loading are very similar to that of EPS in Figure 4a. It exhibits an obvious wide plateau stress. When the proportion of FPUF in the combination material increases, the elasticity modulus and the plateau stress decrease gradually. The cushioning energy absorption of FE-P also decreases with the increase in FPUF proportion for the same deformation, shown in Figure 4b. These are fully consistent with the FE-P stress–strain curves obtained in the model analysis, shown in Figure 4c. For different structure proportions of FPUF in FE-P, the plateau stress of FE-P can be calculated by the equation $\sigma_{P}=\alpha \sigma_{FP}+\left(1-\alpha \right)\sigma_{EP}$. $\sigma_{FP}$ and $\sigma_{EP}$ are the yield stresses of FPUF and EPS, respectively.

**Figure 5:**
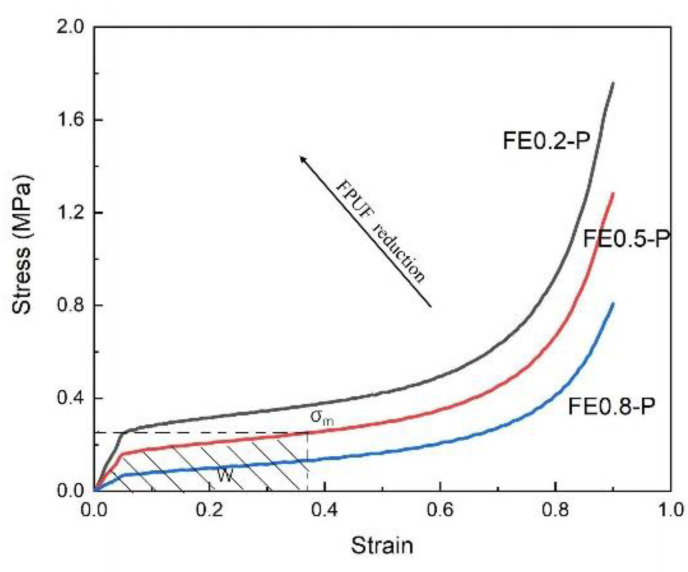
Schematic diagram of FE-P energy absorption.

A superior cushioning material can absorb adequate energy before the deformation enters the compaction phase. For a certain protected product, assuming the maximum stress$\;\sigma_{m}$ and energy absorption *W* are specified in Figure 5. If the plateau stress of cushion material is higher than the$\;\mathrm{stress}\;\sigma_{m}$, high stress will generate and result in damage to the protected product. Compared with traditional cushioning material, adjusting FE-P structural scale parameters can achieve superior cushion effectiveness according to the external impact energy and the permissible stress of the protected product. The different structural scale parameters in FE-P can change the plateau stress of the cushion material. It results in adjusting the peak stress level of the protected product under impact conditions.

The cushioning coefficient *C* is widely used to evaluate the energy absorption performance of cushioning material. It is defined as the ratio of the stress acting on the cushioning material to the impact energy absorbed per unit volume of cushioning material at the stress, expressed as follows.

(6) $$C=\frac{\sigma_{i}}{\int_{0}^{\varepsilon_{i}}\sigma d\varepsilon }$$

Miltz [28,29] proposed to use the *E_ff_* (energy absorption efficiency) to evaluate the energy absorption characteristics of cushioning material.

(7) $$E_{ff}=\frac{\int_{0}^{\varepsilon_{i}}\sigma d\varepsilon }{\sigma_{i}}$$

where$\;\varepsilon_{i}$ and $\sigma_{i}$ are the arbitrary strain and its corresponding stress in the stress–strain curve of cushion material. The formula indicates that energy absorption efficiency (*E_ff_*) is the ratio of the energy absorbed by the cushioning material to the corresponding stress. Energy absorption efficiency is usually used to determine the optimal energy-absorbing working state of a cushioning material for a given impact condition.

**Figure 6:**
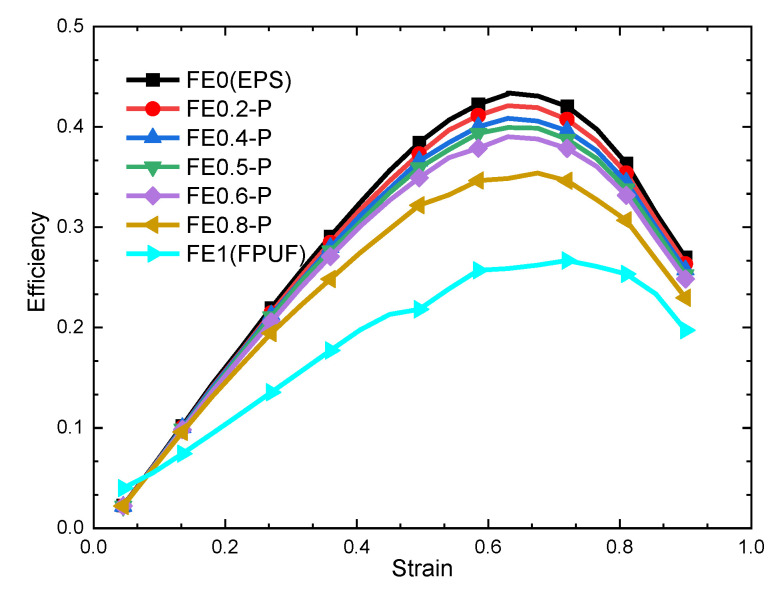
Efficiency–strain curve of FE-P.

The energy absorption efficiency–strain curves of FE-P for different structural scale parameters are shown in Figure 6. It shows that the variation trend of the FE-P energy absorption efficiency curve is consistent with that of EPS but is significantly different from that of FPUF. The energy absorption efficiency of FE-P decreases with the increase in the FPUF ratio. The strain point of the optimal energy absorption efficiency does not change greatly with the structural scale parameter. It means that the change in structural scale parameter can only affect the mechanical properties and cushioning energy absorption capacity of FE-P but does not affect the mechanical deformation mechanism of cushioning absorption of FE-P. It shows that the EPS component plays the dominant role in energy absorption for the FE-P combinations.

**Figure 7:**
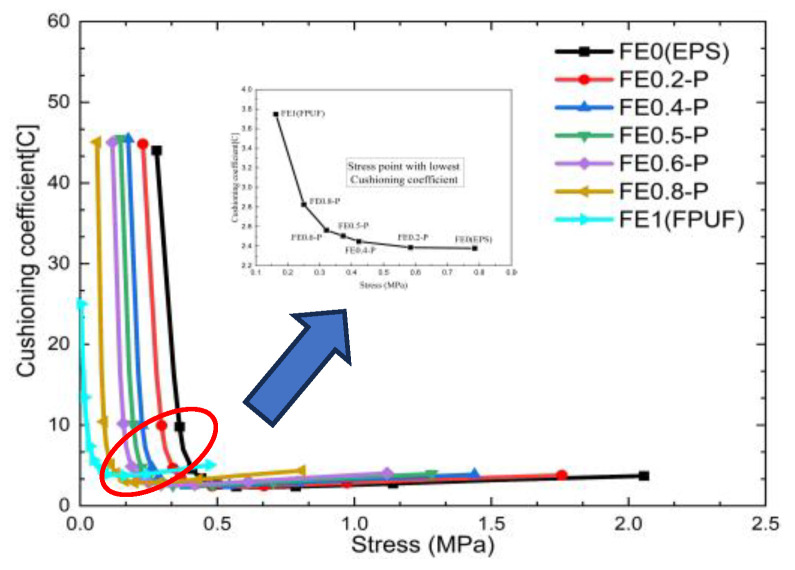
Cushioning coefficient–stress curve of FE-P.

Figure 7 shows the cushioning coefficient–stress curves of FE-P. It shows that the curves decrease sharply with the stress increasing when the stress is less than 0.5 MPa, then increases slowly with stress subsequently. The lowest inflection points represent the stress at the maximum energy absorption of FE-P, which can be taken as the terminal point of the material plateau stress. The positions of the inflection point vary with the proportion of FPUF in FE-P. The characteristic can be utilized to improve impact protection property in the cushion design. The cushion energy absorption of FE-P can be improved by reducing the structural scale parameters of FE-P. It is a possible approach to adjust the stress amplitude transmitted to the protected product under impact conditions.

### 3.2. Cushioning Effectiveness of FE-S

**Figure 8:**
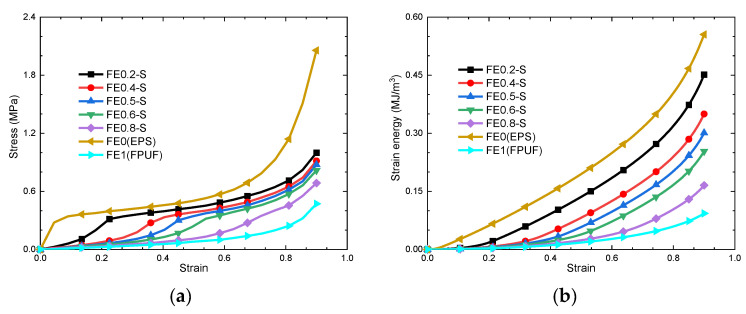
FE-S stress–strain curves (**a**) and strain energy–strain curves (**b**).

Figure 8a shows the stress–strain curves for the FPUF-EPS series combination material under a single uniaxial compressive load. Being different from FPUF and EPS, the curves of the FE-S firstly increase slowly with strain, then increase greatly before entering the stress plateau region. When the combination material enters the compaction region, the stresses increase sharply. The inflection point between the elastic region and stress plateau region represents the yield stress of EPS in FE-S. With the proportion of FPUF in FE-S increasing, the inflection point shifts toward the right. The stress plateau region becomes gradually shorter, and the plateau stress amplitude decreases. When the ratio of FPUF thickness in FE-S is greater than 0.6, there is no obvious stress plateau region. The strain energy absorption is shown in Figure 8b. It indicates that the lower FPUF proportion in FE-S is with higher energy absorption.

**Figure 9:**
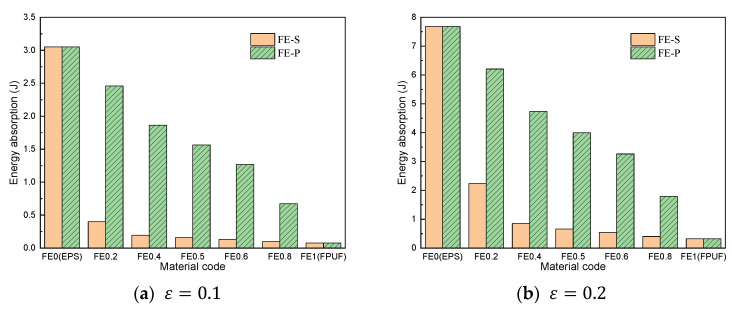

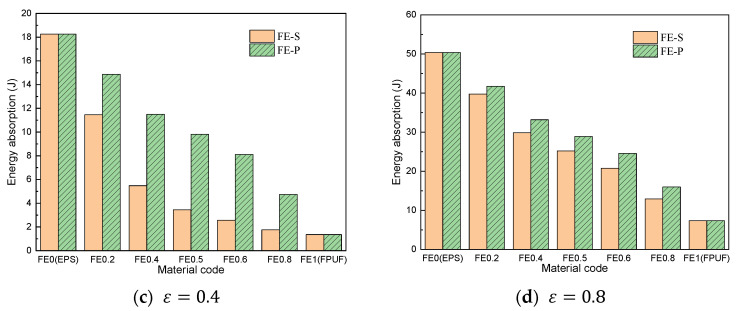
Energy absorption comparison of FPUF-EPS under single compression.

Figure 9 shows the comparison of cushioning energy absorption of FE-P and FE-S with the same structural scale parameters at different strain conditions. The height of the bars represents the cushioning energy absorption of FE-P and FE-S decreases with the FPUF ratio increasing. The cushioning energy absorption of FE-S is smaller than that of FE-P for the same structural scale parameters at given strain conditions. The difference between the cushioning energy absorptions of FE-S and FE-P is more obvious for smaller compressive deformation conditions. It indicates that the cushioning energy absorption capacity of FPUF-EPS with a higher FPUF ratio is weaker than that of FPUF-EPS with a lower FPUF ratio. The cushioning energy absorption capacity of FE-P is much stronger than that of FE-S. The equivalent elastic modulus of the FPUF-EPS combination material in Table 2 indicates that the equivalent elastic modulus of the combination material decreases as the proportion of FPUF increases. Moreover, the equivalent elastic modulus of the FPUF-EPS parallel combination is much larger than that of the FPUF-EPS series combination.

**Figure 10:**
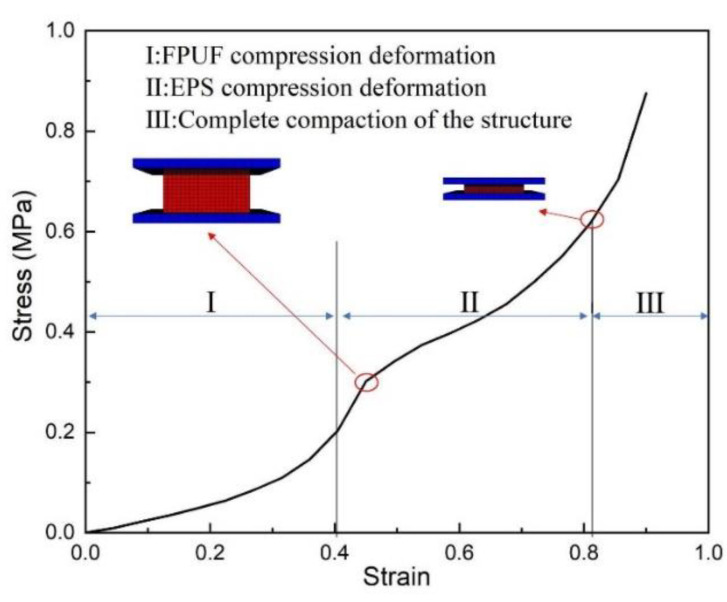
Single compression curve and deformation characteristics of FE0.5-S.

The compression behavior investigated in this paper is the global deformation of the combination material, not involving the local impact deformation and the penetration behavior. The paper aims to analyze the mechanical properties and cushioning effectiveness of the combination materials without the mirco-cellular pores deformation and the evolution process. For example, Figure 10 shows the compression stress–strain curve and deformation process of the combination material (FE0.5-S). There is an obvious small inflection point marked in the curve, which can be taken as the yield stress of EPS. The curve reflects the typical compressive deformation characteristics of the combination material, which can be divided into three stages. Stage I: The FPUF generates large deformation, and the strain increases greatly with stress. Stage II: EPS occurs progressive buckling. The pore wall of EPS bends along the loading direction. The EPS plastic deformation is with energy absorption. Stage III: Both FPUF and EPS are compacted, and the whole combination structure is densified fully.

**Figure 11:**
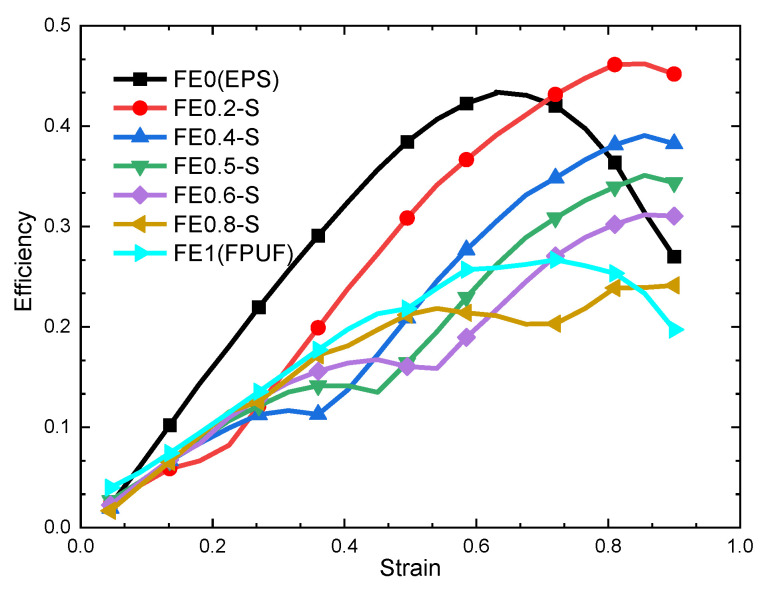
Efficiency–strain curve of FE-S.

Based on the energy absorption efficiency–strain curve of FE-S in Figure 11, it can be seen that the variation pattern of cushioning energy absorption efficiency versus strain of FE-S is different from those of FE-P (in Figure 6), FPUF, and EPS. A clear trough appears in the curve of FE-S, which is the connection point between stage I and stage II. The cushioning energy absorption efficiency of FE-S is relatively small and increases slowly with strain in stage I. It goes up sharply with strain increasing in stage II and rises higher than those of FE-P, FPUF, and EPS in stage III. It indicates that FE-S has a higher maximum cushioning energy absorption efficiency and larger cushioning energy absorption strain.

**Figure 12:**
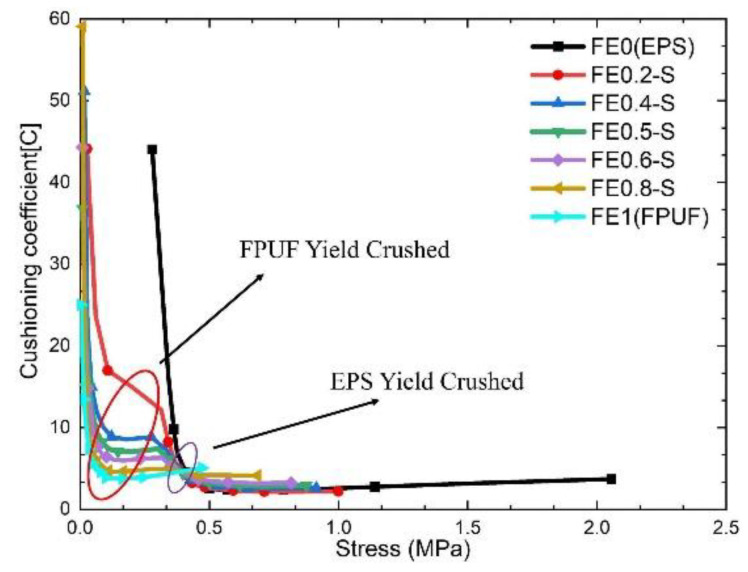
Cushioning coefficient–stress curve of FE-S.

The cushioning coefficient–stress curves of FE-P, FPUF, and EPS have only one peak in Figure 7, while the cushioning coefficient–stress curve of FE-S has two peaks in Figure 12. According to the compressive deformation characteristics of FE-S material, the two stress peaks represent the yield crush points of FPUF and EPS, respectively. The stress of FPUF at the minimum cushioning coefficient is close to the first ultimate stress of FE-S. The cushioning coefficient increases with the structural scale parameter decreasing in the process (stage I). The stress of EPS at the minimum cushioning coefficient is close to the second ultimate stress of FE-S. The cushioning coefficient increases with the structural scale parameter increasing in the process (stage II). For FE-P, FPUF, and EPS, there is plateau stress in the stress–strain curves. The plateau stress of FE-P can be adjusted by changing the structural scale parameter. However, the stress–strain curve of FE-S is with two plateau stresses. The lower plateau stress depends on the yield stress of FPUF, and the higher plateau stress depends on the yield stress of EPS. So, the cushion pattern can be designed according to the protected object shock resistance property and the impact conditions.

## 4. Cushioning Effectiveness of FPUF-EPS under Single-Impact Loading

In order to investigate the cushioning behavior of FE-P and FE-S under a single-impact load, several impact cases are provided in the following numerical simulation. The impact plate mass is 1 kg, and the initial velocities are 3 m/s (4.5 J), 5 m/s (12.5 J), and 10 m/s (50 J) in the simulations, respectively. Thirty-six simulation cases are carried out considering combination materials in parallel and series with different combination ratios and different initial impact velocities.

### 4.1. Force–Time Response Characteristics of FPUF-EPS

**Figure 13:**
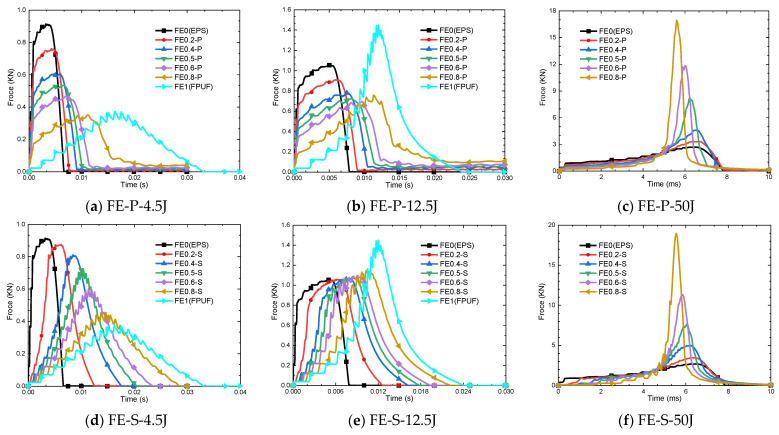
Force–time curve of FPUF-EPS combination materials under impact loads.

The force–time curve is a traditional criterion to evaluate the cushioning performance of the material under impact loads. Superior cushion material usually exhibits stable stress and long plateau durations. Figure 13 shows the force–time curves of FE-P and FE-S with different combination ratios under three impact conditions of 4.5 J, 12.5 J, and 50 J. As the impact case of 50 J exceeds the energy absorption limit of FPUF, the force–time curves of FE1 (FPUF) are not provided in Figure 13c,f.

For the impact condition of 4.5 J in Figure 13a, the FE0 (EPS) material exhibits the maximum peak force of 0.91 kN and the shortest impact duration of 6.6 ms. The FE1 (FPUF) exhibits the smallest peak force of 0.37 kN and the longest impact duration of 33.0 ms. For the impact condition of 12.5 J in Figure 13b, FE0 (EPS) exhibited the second-highest peak force of 1.06 kN and the shortest impact duration of 7.9 ms. The FE1 (FPUF) exhibited the maximum peak force of 1.45 kN and the longest impact duration of 25.0 ms. The FE0 (EPS) exhibits the minimum peak force of 2.69 kN and the shortest impact duration of 7.8 ms under the impact condition of 50 J. As the impact energy of 50 J is higher than the energy absorption limit of FE1 (FPUF), the force–time curve cannot be provided by numerical simulation, and it should exhibit the maximum peak force and the shortest impact duration. The results indicate that the external impact energy has a great influence on the cushioning performance of FE0 (EPS) and FE1 (FPUF) under impact compression loading. For low-impact energy conditions, FE1 (FPUF) has a much better cushioning performance than that of FE0 (EPS). In contrast, FE0 (EPS) has better cushioning performance for high-impact energy conditions.

The characteristics induce the peak force of FE-P and FE-S to decrease with combination scale parameters increasing, and the impact duration becomes gradually longer under impact conditions of 4.5 J and 12.5 J. For the impact condition of 4.5 J in Figure 13a,d, the peak force of FE0.8-P is about 60% of FE0 (EPS), and the impact duration is about 2.4 times that of FE0 (EPS). The peak force of FE0.8-S is about 48% of FE0 (EPS), and the impact duration is about 3.5 times that of FE0 (EPS). For the impact condition of 12.5 J in Figure 13b,e, the peak force of FE0.8-P is about equal to that of FE0 (EPS), and the impact duration is about 1.8 times that of FE0 (EPS). The peak force of FE0.8-S is about 48% of FE0 (EPS), and the impact duration is about 2.8 times that of FE0 (EPS). It indicates that the impact velocity (impact energy) affects the peak force and impact duration. The FPUF-EPS structures of the combination scale parameters behave in different mechanical responses. So, the FPUF-EPS combination pattern can be designed to obtain an acceptable mechanical environment under various impact conditions.

### 4.2. Mechanical Response Characteristics of FPUF-EPS

**Figure 14:**
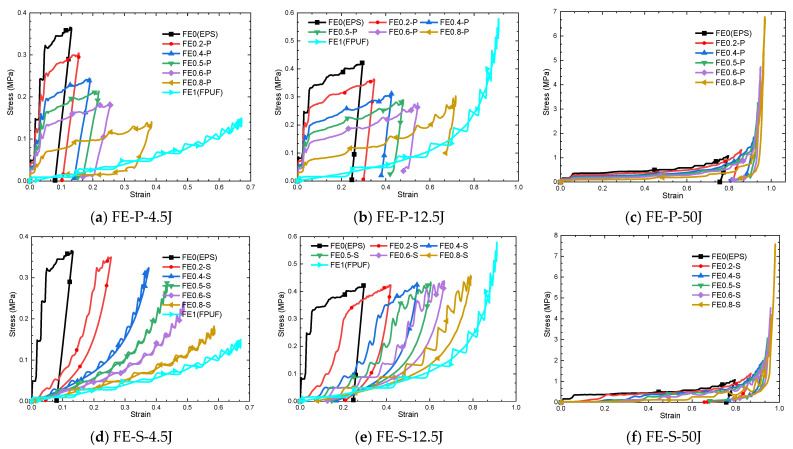
Stress–strain curve of FPUF-EPS combination materials under impact loads.

The stress–strain curves of FE-P and FE-S with different combination ratios under three impact conditions are shown in Figure 14. The material model of FE1 (FPUF) is taken as a hyperfoam, which makes the stress–strain curves of loading and unloading almost overlap. The stress–strain curves of FE-P and EPS under impact compression loading exhibit wide plateau stress characteristics, and the curves of the loading and the unloading are parallel. The maximum peak stress and the residual strain gradually increase with the impact velocity. With the FPUF ratio increasing, the stress plateau decreases, and the plateau width increases. It indicates that the proportion of FPUF increasing can reduce the peak stress of the combination materials by increasing the plastic deformation. FE-P material with a larger FPUF ratio occurs easily during densification under high-velocity impact conditions.

The stress–strain curves of the FE-S material show completely different shapes under different energy impact conditions. For the impact condition of 4.5 J in Figure 14d, the stress–strain curve gradually changes from square to linear with the increasing FPUF ratio. The maximum strain increases with the FPUF ratio, and the peak stress and residual strain decrease with the FPUF ratio. It indicates that the FPUF and the EPS have load-supporting capacity in the loading process, but the EPS loses load-supporting capacity, and only the FPUF retains the capacity in the unloading process. The stress–strain curves of FE-S also exhibit wide plateau stress in the impact condition of 12.5 J in Figure 14e. The plateau stress changes little for different FPUF ratio conditions. But, the proportion of FPUF in FE-S greatly affects the maximum strain and the residual strain. When the impact condition increases to 50 J in Figure 14f, the FE-S is fully compacted.

**Figure 15:**
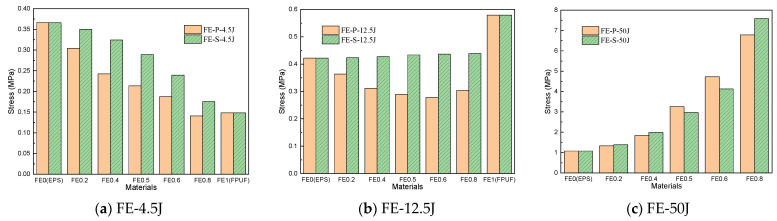
Maximum stress comparison of FPUF-EPS combination materials under impact loads.

Based on the simulation analysis, the mechanical properties of FE-P and FE-S are different in impact compression conditions. For the FE-P combination, the FPUF and the EPS undertake the loading together. The proportion of FPUF increasing brings the peak stress of FE-P decreasing. For the FE-S combination, the FPUF and the EPS have identical loading, but the FPUF occurs in the larger strain. So, FE-S behaves with a larger peak stress than that of FE-P at low and medium energy impact conditions but smaller peak stress at high-impact conditions, as shown in Figure 15. The FPUF ratio increasing in FE-S can only induce the deformation increase in the combined material but not weaken the peak stress of the combined material. Being different from FE-P, the residual strain of FE-S is greatly reduced with the FPUF ratio increasing, which allows FE-S to have more buffering space in multiple impact loading conditions.

### 4.3. Energy Absorption and Influencing Factor of FPUF-EPS under Single-Impact Loading

Table 3 shows the cushioning energy absorption of the FPUF-EPS combination material under single-impact compression loading. For the same energy impact condition, the cushioning energy absorption of FE-P and FE-S gradually decreases with the ratio of FPUF increasing. The cushioning energy absorption of FE-S is smaller than that of FE-P when the structural scale parameters of FE-P and FE-S are identical. It indicates that the energy absorption of a series combination is more weakened than that of a parallel combination.

**Figure 16:**
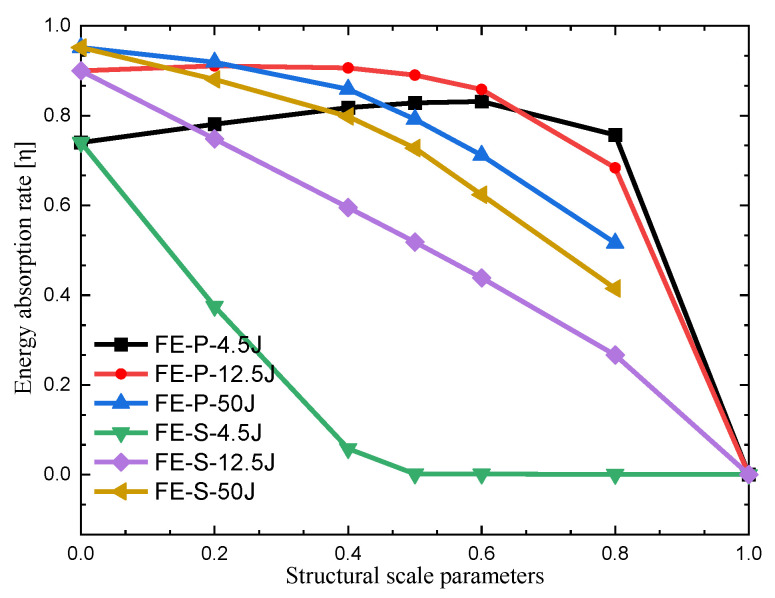
Comparison of energy absorption rate of FPUF-EPS combination materials under impact loading.

The cushioning energy absorption rate can be used to evaluate the cushioning effectiveness of the FPUF-EPS combination material under impact compression loading, which is defined as the ratio of the cushioning energy absorption to the impact energy, expressed as η. Figure 16 shows the cushioning energy absorption rate of the FPUF-EPS combination material under 4.5 J, 12.5 J, and 50 J impact conditions.

Figure 16 shows that EPS (FE0) has a high cushioning energy absorption rate, which is higher than that of FPUF (FE1). For the identical structural scale parameter of FE-S, the energy absorption rate increases gradually with the impact energy. With the proportion of FPUF increasing, the cushioning energy absorption rate decreases gradually. The main cushioning energy of FE-S is absorbed by the elastic deformation of the FPUF in the small loading condition, and the EPS dissipates much impact energy in the densification deformation stage. The more cushioning energy absorbed by the FPUF part of the combined material, the lower the cushioning energy absorption rate of FE-S. Being different from FE-S, the FPUF and EPS of FE-P are with identical deformation in the impact process. The increase in the FPUF ratio will induce the energy absorption capacity of FE-P to decrease. The cushioning energy absorption rate of FE-S is between that of EPS and FPUF. It increases with the impact energy and the ratio of EPS. The cushioning energy absorption rate of FE-P first increases and then decreases with the structural scale parameter increasing in 4.5 J and 12.5 J impact conditions.

## 5. Cushioning Effectiveness of FPUF-EPS under Multiple Compression Loadings

The compression reversible characteristics of FPUF and the compressive non-reversible characteristics of EPS induce the residual strains of the two materials to be different after loading and unloading, which also affects the cushioning energy absorption performance of the two materials in multiple loading conditions. FE-P and FE-S show different deformation behavior under compression loading. It brings FE-P and FE-S with different combination ratios that exhibit completely different mechanical properties and cushioning effectiveness under multiple compression loadings.

### 5.1. Cushioning Effectiveness of FE-P under Multiple Compression Loadings

**Figure 17:**
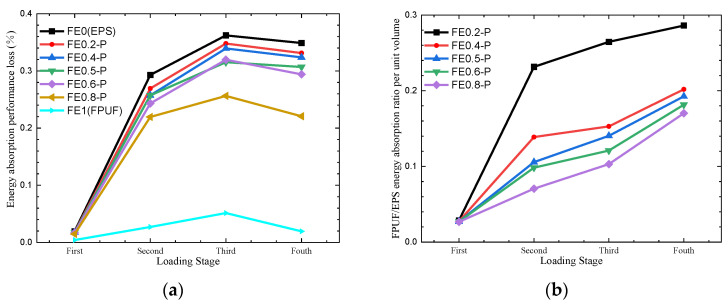
Energy absorption performance loss (**a**) and FPUF/EPS Energy absorption ratio per unit volume (**b**) of FE-P.

Numerical simulations are performed for the FPUF-EPS combination material under multiple compressive loadings. The loading condition is the boundary displacement controlling. The multiple compressive loadings are applied by four loading steps. The deformation is compressed to 0.1 and then released in the first loading step. The deformation strain of 0.2, 0.4, and 0.8 is applied and released in the subsequent loading steps, respectively. Table 4 shows the force comparisons of the different FE-P combination materials at identical strain under single and multiple compression loadings. It shows that the forces are a little different under single and multiple loading conditions, and the stresses in FE-P are not greatly affected by loading history. Table 5 shows the energy absorption comparison of FE-P material under single and multiple compression loadings. Compared with the energy absorption of FE-P under a single compression loading in Figure 9, each step of multiple loadings is with energy dissipation caused by EPS plastic deformation. The structure scale parameter of FE-P (FPUF ratio) increasing induces the energy absorption performance loss decrease in Figure 17a. The ratio of the energy absorption by FPUF to that by EPS is shown in Figure 17b. It decreases with the structural scale parameter increasing but increases with the loading time increasing. It indicates that FPUF can improve the cushioning effectiveness of FE-P under multiple loadings.

### 5.2. Cushioning Effectiveness of FE-S under Multiple Compression Loadings

**Figure 18:**
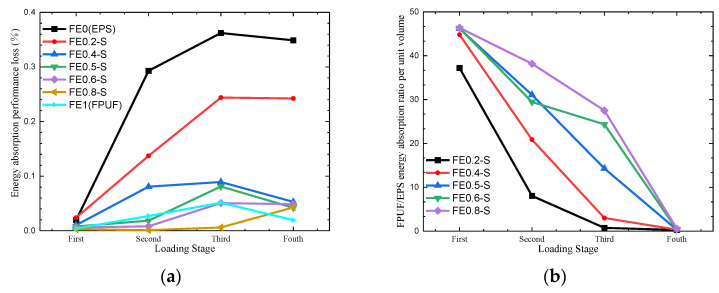
Energy absorption performance loss (**a**) and FPUF/EPS energy absorption ratio per unit volume (**b**) of FE-S.

The force comparison of FE-S material under single and multiple compression loadings at identical strain is shown in Table 6. Being similar to FE-P in Table 4, the difference in force peak between single and multiple loading conditions is not obvious. It indicates that the loading history does not greatly affect the stress. Table 7 shows the cushioning energy absorption of FE-S in the four loading conditions. Compared with the energy absorption of FE-S under a single compression loading in Figure 9, each step of the multiple loadings is with energy dissipation caused by EPS plastic deformation. For the same loading course, the energy absorbed by FE-S at each loading step is smaller than that of FE-P. The loss of cushioning energy absorption performance under multiple loading of FE-S is much smaller than that of FE-P and EPS in Figure 17 and Figure 18. When FE-S is loaded by multiple small compressive loadings, the most cushioning energy absorption is stored by FPUF elastic deformation. The proportion of FPUF in FE-S increases induces the cushioning performance loss decrease in Figure 18a. The ratio of the energy absorption by FPUF to that by EPS is shown in Figure 18b, which increases with the structural scale parameter increasing but decreases with the loading time increasing. It is different from that of FE-P in Figure 17b. The ratio of energy absorption by FPUF in FE-S is much higher than that in FE-P. It means the proportion of elastic deformation energy is higher in FE-S.

## 6. Conclusions

(1) The mechanical behavior and cushioning performance of FE-P and FE-S under single quasi-static compression loading are different. The plateau stress cushioning energy absorption of material combination changes linearly with the FPUF ratio. It means the cushioning energy absorption and the stress transmitted to the protection products can be adjusted by the structural scale parameters of the material combination.
(2) FE-P and FE-S have different mechanical response behavior under impact conditions; the cushioning energy absorption of FE-S is lower than that of FE-P for the same deformation. The cushioning energy absorption rate of FE-S is positively related to the EPS ratio and the impact energy. The cushioning energy absorption rate of FE-P first increase and then decrease with the structural scale parameter increasing under the same impact condition. FE-P material is suitable for stress-sensitive protection products, and FE-S is suitable for acceleration-sensitive protection products.
(3) FPUF is with small energy absorption but without cushioning energy absorption capacity loss in multiple compression loading conditions. EPS has high cushioning energy absorption but cannot maintain cushioning properties after multiple impact loading. For the FE-P and FE-S combination, the larger the proportion of FPUF, the smaller the cushioning energy absorption loss of FPUF-EPS. The proportion of EPS increases brings the stronger cushioning energy absorption of FPUF-EPS. So, the FE-P combination is superior for high-strength impact protection, and the FE-S combination is superior for low-strength impact conditions.

## Figures and Tables

**Table 1:** FPUF-EPS combination materials.

Materials Type	FE0 (EPS)	FE0.2	FE0.4	FE0.5	FE0.6	FE0.8	FE1 (FPUF)
FPUF size (mm)	0	50 × 50 × 10	50 × 50 × 20	50 × 50 × 25	50 × 50 × 30	50 × 50 × 40	50 × 50 × 50
EPS size (mm)	50 × 50 × 50	50 × 50 × 40	50 × 50 × 30	50 × 50 × 25	50 × 50 × 20	50 × 50 × 10	0
Equivalent density (kg/m^3^)	43.5	50.4	57.2	60.7	64.1	70.9	77.8

**Table 2:** Equivalent elastic modulus of the FPUF-EPS combination materials.

Material	FE0 (EPS)	FE0.2-P	FE0.4-P	FE0.5-P	FE0.6-P	FE0.8-P
Equivalent Elastic Modulus	6.19	5	3.79	3.18	2.57	1.34
Material	FE0.2-S	FE0.4-S	FE0.5-S	FE0.6-S	FE0.8-S	FE1 (FPUF)
Equivalent Elastic Modulus	0.65	0.34	0.27	0.22	0.16	0.11

**Table 3:** Energy absorption of FPUF-EPS combination materials under single-impact loads.

Materials	Structural Scale Parameters (Energy Absorption/J)						
	FE0 (EPS)	FE0.2	FE0.4	FE0.5	FE0.6	FE0.8	FE1 (FPUF)
FE-P-4.5J	3.3	3.5	3.7	3.7	3.7	3.4	1.7 × 10^−3^
FE-S-4.5J	3.3	1.7	0.3	7.5 × 10^−3^	6.0 × 10^−3^	3.8 × 10^−3^	1.7 × 10^−3^
FE-P-12.5J	11.2	11.4	11.3	11.1	10.7	8.5	7.8 × 10^−3^
FE-S-12.5J	11.2	9.4	7.4	6.5	5.5	3.3	7.8 × 10^−3^
FE-P-50J	47.6	46.0	42.9	39.6	35.6	25.8	---
FE-S-50J	47.6	44.0	39.9	36.4	31.2	20.7	---

**Table 4:** Force comparison of FE-P material under single and multiple compression loadings.

Materials	FE0.2-P		FE0.4-P		FE0.5-P		FE0.6-P		FE0.8-P	
Strain	Multiple (N)	Single (N)	Multiple (N)	Single (N)	Multiple (N)	Single (N)	Multiple (N)	Single (N)	Multiple (N)	Single (N)
0.1	703.4	703.4	536.5	536.5	454.6	454.6	371.3	371.3	204.3	204.3
0.2	808.5	790.4	569.5	610.1	504.5	516.2	452.0	425.9	247.4	250.3
0.4	967.5	951.1	708.0	749.6	633.0	648.4	550.5	547.6	340.5	350.2
0.8	2302.3	2311.5	1910.2	1883.2	1651.6	1669.6	1476.0	1455.3	1031.8	1035.5

**Table 5:** Energy absorption comparison of FE-P material under single and multiple compression loadings.

Loading Stage	Structural Scale Parameters (Energy Absorption/J)						
	FE0 (EPS)	FE0.2-P	FE0.4-P	FE0.5-P	FE0.6-P	FE0.8-P	FE1 (FPUF)
Strain 0.1	3.0	2.4	1.8	1.5	1.2	0.7	8.0 × 10^−2^
Strain 0.2	5.4	4.5	3.5	3.0	2.5	1.4	0.3
Strain 0.4	11.6	9.7	7.6	6.7	5.5	3.5	1.4
Strain 0.8	32.8	27.9	22.4	20.0	17.4	12.5	7.2
Multiple sum	52.8	44.6	35.4	31.2	26.6	18.0	9.0
Single	50.4	41.8	33.2	28.9	24.6	16.0	7.4

**Table 6:** Force comparison of FE-S material under single and multiple compression loadings.

Materials	FE0.2-S		FE0.4-S		FE0.5-S		FE0.6-S		FE0.8-S	
Strain	Multiple (N)	Single (N)	Multiple (N)	Single (N)	Multiple (N)	Single (N)	Multiple (N)	Single (N)	Multiple (N)	Single (N)
0.1	177.1	176.6	79.8	79.4	63.8	62.4	54.8	52.1	39.4	41.9
0.2	622.0	623.2	179.1	189.1	126.6	139.3	78.6	114.7	83.3	79.2
0.4	1038	983.9	869.5	817.8	504.3	493.6	314.8	317.1	149.9	196.4
0.8	2242	1743.9	1875	1586.8	1677	1505.1	1480	1396.1	1115	1106.5

**Table 7:** Energy absorption comparison of FE-S material under single and multiple compression loadings.

Loading Stage	Structural Scale Parameters (Energy Absorption/J)						
	FE0 (EPS)	FE0.2-S	FE0.4-S	FE0.5-S	FE0.6-S	FE0.8-S	FE1 (FPUF)
Strain 0.1	3.0	0.4	0.2	0.2	0.1	0.1	8.0 × 10^−2^
Strain 0.2	5.4	1.9	0.8	0.7	0.5	0.4	0.3
Strain 0.4	11.6	11.0	5.0	3.2	2.4	1.8	1.4
Strain 0.8	32.8	30.1	28.3	24.1	19.8	12.4	7.2
Multiple sum	52.8	43.5	34.2	28.1	22.9	14.6	9.0
Single	50.4	39.8	29.9	25.2	20.8	12.9	7.4

## Data Availability

The data presented in this study are available on request from the corresponding author.

## Abbreviations

**Symbols and Acronyms**	**Explanations**
FPUF	Flexible polyurethane foam
EPS	Expanded polystyrene foam
FE-P	FPUF-EPS parallel combination material
FE-S	FPUF-EPS series combination material
FE0	Neat EPS material (Thickness of EPS is 50 mm)
FE0.2-P	FPUF-EPS parallel combination material (Thickness of FPUF is 10 mm, EPS is 40 mm)
FE0.2-S	FPUF-EPS series combination material (Thickness of FPUF is 10 mm, EPS is 40 mm)
FE0.4-P	FPUF-EPS parallel combination material (Thickness of FPUF is 20 mm, EPS is 30 mm)
FE0.4-S	FPUF-EPS series combination material (Thickness of FPUF is 20 mm, EPS is 30 mm)
FE0.5-P	FPUF-EPS parallel combination material (Thickness of FPUF is 25 mm, EPS is 25 mm)
FE0.5-S	FPUF-EPS series combination material (Thickness of FPUF is 25 mm, EPS is 25 mm)
FE0.6-P	FPUF-EPS parallel combination material (Thickness of FPUF is 30 mm, EPS is 20 mm)
FE0.6-S	FPUF-EPS series combination material (Thickness of FPUF is 30 mm, EPS is 20 mm)
FE0.8-P	FPUF-EPS parallel combination material (Thickness of FPUF is 40 mm, EPS is 10 mm)
FE0.8-S	FPUF-EPS series combination material (Thickness of FPUF is 40 mm, EPS is 10 mm)
FE1	Neat FPUF material (Thickness of FPUF is 50 mm)
